# *Aspergillus fumigatus* Endophthalmitis with Necrotizing Scleritis following Pars Plana Vitrectomy

**DOI:** 10.1155/2016/9289532

**Published:** 2016-06-09

**Authors:** Anna M. Gruener, Felicity Allen, Miles R. Stanford, Elizabeth M. Graham

**Affiliations:** The Medical Eye Unit, St. Thomas' Hospital, Guy's and St. Thomas' NHS Foundation Trust, London SE1 7EH, UK

## Abstract

We present a case of* Aspergillus fumigatus* endophthalmitis complicated by necrotizing scleritis in a 68-year-old man with diet-controlled diabetes, after retinal detachment repair. He was initially treated with systemic steroids for surgically induced necrotizing scleritis following routine pars plana vitrectomy. An additional diagnosis of endophthalmitis was made when the patient developed a hypopyon. Repeat vitreous culture isolated* Aspergillus fumigatus*. Symptoms improved following antifungal treatment leaving the patient with scleromalacia and an advanced postoperative cataract. Fungal scleritis and endophthalmitis are rare complications of intraocular surgery with sight-threatening consequences, and, as this case demonstrates, may even occur concomitantly. The overlapping features of both conditions can make differentiating one from the other difficult. A fungal aetiology should be considered in cases of postoperative scleritis and endophthalmitis that are protracted and refractory to standard therapy. Even in cases of early diagnosis and treatment, visual outcomes in* Aspergillus* endophthalmitis and scleritis are variable and often disappointing, not infrequently necessitating enucleation of a painful blind eye.

## 1. Introduction

Exogenous fungal endophthalmitis is a rare complication of fungal keratitis, penetrating trauma, and intraocular surgery. The most common isolates in postoperative fungal endophthalmitis are* Aspergillus* spp., which can be challenging to treat [1]. We describe a unique case of exogenous* Aspergillus* endophthalmitis complicated by necrotizing scleritis after routine pars plana vitrectomy.

## 2. Case Report

**Figure 1:**
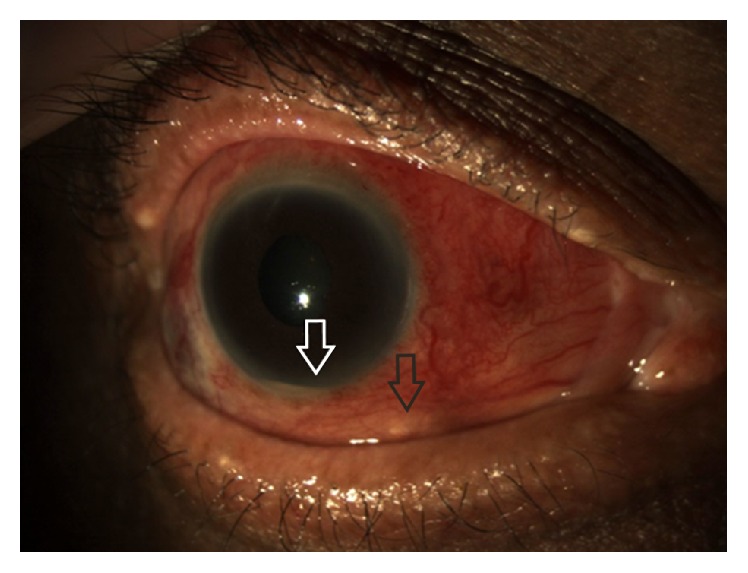
Anterior segment photograph of the right eye showing an inferonasal conjunctival abscess (black arrow) and hypopyon (white arrow) with marked conjunctival injection and chemosis.

**Figure 2:**
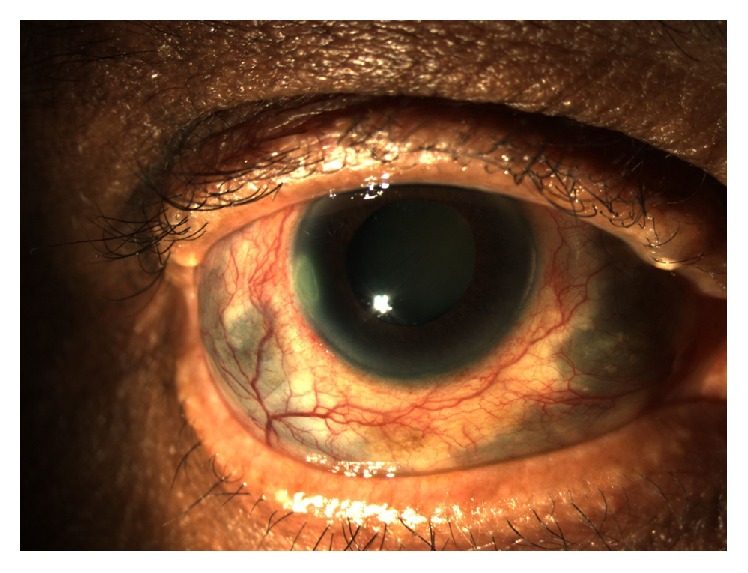
Anterior segment photograph of the right eye showing reduced conjunctival injection and increasingly prominent areas of scleromalacia following treatment with antifungals.

A 68-year-old diet-controlled diabetic man underwent uneventful three-port 23-gauge pars plana vitrectomy with fluid-air exchange, sulfur hexafluoride 20% gas tamponade, and cryoretinopexy for a right primary macula-on rhegmatogenous retinal detachment at an outside provider. Twelve days post-operatively he complained of severe ocular pain. He was diagnosed with surgically induced necrotizing anterior and posterior scleritis with choroidal effusions. He was treated with increasing doses of oral Prednisolone (initially 40 mg, q.d., then 60 mg, q.d., and eventually 80 mg, q.d.) to no effect. Subsequent treatment with intravenous methylprednisolone (IVMP) pulse therapy at 1 g/d for three days resulted in partial pain relief and development of a hypopyon. He was referred to our unit where a tentative diagnosis of post-operative bacterial endophthalmitis was made. His visual acuity (VA) at this stage was 20/60 OD. Vitreous aspiration was performed and intravitreal injections of vancomycin (2 mg/0.1 mL) and ceftazidime (2 mg/0.1 mL) were administered. Oral Moxifloxacin (400 mg, q.d.) was started and oral steroids (40 mg, q.d.) continued. After two days, the hypopyon contracted and VA improved to 20/40. Four weeks later, the patient presented with a recurrent hypopyon and conjunctival abscess inferonasally (Figure 1). Needle aspiration of the abscess, anterior chamber tap, and repeat vitreous aspiration were performed. Repeat intravitreal injections of vancomycin (2 mg/0.1 mL) and ceftazidime (2 mg/0.1 mL), in addition to amphotericin B (10 mcg/0.1 mL), were administered. Gram stain from the conjunctival aspirate showed* Pseudomonas aeruginosa* and culture of the repeat vitreous sample isolated* Aspergillus fumigatus*. The patient received one dose of intravenous amphotericin B (100 mg) and piperacillin/tazobactam (4 g/0.5 g), followed by a four-week course of oral ciprofloxacin (500 mg, b.i.d.) and voriconazole (400 mg, b.i.d.); oral steroids were tapered further. The right eye developed scleromalacia and an advanced post-vitrectomy cataract with a VA of counting fingers (Figure 2). The patient's pain ceased entirely and the eye has been quiescent to date.

## 3. Discussion

Although uncommon,* Aspergillus* is known to cause endogenous endophthalmitis in the immunocompromised and less commonly the immunocompetent. Exogenous* Aspergillus* endophthalmitis is even rarer, but has been identified as the most common form of post-operative fungal endophthalmitis [1]. Post-operative* Aspergillus* endophthalmitis has been reported in the context of cataract surgery, penetrating keratoplasty, and blebitis. A detailed search of PubMed reveals this to be the first case of exogenous* Aspergillus* endophthalmitis following vitrectomy.

Endogenous* Aspergillus* endophthalmitis may feature prominent yellowish exudative chorioretinal infiltrates with invasion of retinal and choroidal vessel walls [2]. In contrast, exogenous* Aspergillus* endophthalmitis may show significant hypopyon and vitritis, with sparing of the retina [3, 4]. Culture of vitreous aspirate, in conjunction with examination of Gram- or Giemsa-stained smears, will identify the organism in the majority of cases. In addition, quantitative broad-range real-time PCR is a novel diagnostic tool in diagnosing fungal, and, in particular,* Aspergillus* endophthalmitis [5]. Our patient developed infection in a vitrectomized eye, which reduced the likelihood of a positive microbiology result. However, we were eventually able to culture* Aspergillus* from a repeat vitreous sample. Vitreous biopsy should be combined with intravitreal injection of amphotericin B or voriconazole, which may be combined with pars plana vitrectomy. Voriconazole has excellent oral bioavailability and intraocular penetration and is generally given for at least a month, although no clear guidance exists. The role of corticosteroids in the management of* Aspergillus* endophthalmitis remains unclear. As our patient's fungal endophthalmitis was preceded and complicated by anterior necrotizing and posterior scleritis, systemic high-dose steroids were administered for pain control, which, together with his diabetes, may have promoted and protracted fungal growth. Whether the patient's scleritis was primarily fungal in nature or surgically induced is impossible to tell. Therefore, scleritis after intraocular surgery should raise concerns about an infectious process. Fungal infection should always be considered in the differential diagnosis, especially if there is an inappropriate response to intravitreal antibiotics. Occasionally, multiple vitreous biopsies and/or a diagnostic pars plana vitrectomy may be needed to make the diagnosis.

As far as final outcomes are concerned, even in cases of early diagnosis and treatment of* Aspergillus* endophthalmitis, visual results are variable and often disappointing, not infrequently necessitating enucleation of a painful blind eye.
